# Synthesis and Characterization of Cement Clinker Using Waste Glass and Oyster Shells as Natural Ore Substitutes

**DOI:** 10.3390/ma17235980

**Published:** 2024-12-06

**Authors:** Tao Yang, Yajun Wang, Yang Bai, Xiaoyang Chen

**Affiliations:** School of Maritime and Civil Engineering, Zhejiang Ocean University, Zhoushan 316000, China; yangtao@zjou.edu.cn (T.Y.); baiyang@zjou.edu.cn (Y.B.); chenxiaoyang@zjou.edu.cn (X.C.)

**Keywords:** recycled powdered glass (RPG), oyster shell residue (OSHL), RPG and OSHL ecological cement (ROEC), cement clinker

## Abstract

This study investigates the process of synthesizing eco-cement clinker using recycled powdered glass (RPG) and oyster shell residue (OSHL) as primary raw materials. Analysis of the mineral composition of RPG revealed that it primarily consists of silica and contains a high level of alkali metal oxides, while OSHL comprises a high-purity trigonal calcite structure, similar in chemical composition to limestone. Comparative analysis of the synthesized ecological cement and its hydration products showed that, after heat treatment at 1200 °C, the alkali metal content in the H12 sample significantly decreased, thereby meeting the standards for cement raw materials. The hydration process of RPG and OSHL ecological cement (ROEC) exhibited greater reactivity, with the presence of alkali ions (such as Na⁺ and K⁺) accelerating the cement hydration reaction and significantly enhancing early strength. Furthermore, as the curing time increased, the hydration products of ROEC became more uniform, ultimately consisting primarily of calcite. These findings indicate that the combination of RPG and OSHL offers a novel approach to cement synthesis, while reducing the content of alkali metal oxides, thereby aligning the cement clinker more closely with modern production standards.

## 1. Introduction

With the rapid consumption of global resources, the widespread use of cement-based materials poses a serious challenge to the sustainability of natural resources. Excessive reliance on traditional natural mineral raw materials not only accelerates resource depletion but also triggers a series of environmental issues, including ecological damage from mineral extraction and increased carbon emissions [1]. Over the past near-century, the global usage of cement has experienced significant growth, driven primarily by infrastructure development, accelerated urbanization, and increased demand in the construction industry. As of 2019, the global production of cementitious materials reached approximately 4.1 billion tons [2], with projections indicating that this figure will increase 2.5 times by 2050, predominantly in developing countries [3]. However, the production of 1 ton of cementitious material requires the consumption of 1.7 tons of mineral raw materials, including limestone, sandstone, clay, and iron slag, which inevitably exacerbates resource depletion and leads to severe environmental problems [4]. Therefore, utilizing waste glass and oyster shells as substitutes for traditional natural minerals in cement-based materials presents significant resource conservation and environmental protection benefits [5].

Waste glass, particularly sodium-calcium silicate glass from everyday recycling, shares certain chemical similarities with the clay used in cement manufacturing, especially regarding its silica content. As a result, research has explored the use of waste glass as a partial substitute for clay components in cement production. Studies indicate that incorporating waste glass can promote the formation of liquid phases within the temperature range of 950 °C to 1250 °C. However, this addition can also lead to a reduction in the C3S content of the clinker while increasing the C3A content, which may result in the rapid setting and poor early strength development of the cement [6].

Thus, while waste glass theoretically has the potential to replace some clay raw materials, careful control of the alkali oxide content in the glass is crucial in practical applications to mitigate any adverse effects on cement performance. Research suggests that a high alkali content in glass, particularly Na_2_O, can lead to alkali volatilization during the firing process, which not only affects the formation of clinker minerals but may also cause production issues such as ring formation in the kiln [4]. Furthermore, during the hydration of cement, the presence of a high alkali content may initially enhance early strength; however, this may be followed by a decline in strength beyond 28 days. Additionally, high-alkali cement can induce alkali–aggregate reactions (AAR) in concrete, posing significant long-term durability challenges for high-alkali cement [7].

With the rapid development of aquaculture, the substantial production of oyster shells as a byproduct has resulted in serious environmental pollution issues. In recent years, oyster shells, due to their high calcium carbonate (CaCO_3_) content, have emerged as a potential substitute for limestone. Some studies have indicated that, following appropriate pretreatment, oyster shells can successfully replace limestone in the synthesis of cement clinker, producing key minerals such as C3S and C2S that are similar to those found in traditional clinker [8]. Moreover, using oyster shells as a substitute for limestone can reduce the consumption of natural resources and yield lower production costs.

Compared to waste glass, the primary advantage of using oyster shells in cement clinker production lies in their chemical composition, which is closer to that of limestone, thus avoiding the high-alkalinity issues associated with waste glass. Despite the promising applications of both waste glass and oyster shells in cement production, existing research suggests that they may present different technical challenges during the clinker synthesis process. For instance, the high alkali content of waste glass can lead to rapid setting and strength issues, whereas oyster shells, owing to their similarity to limestone in terms of chemical composition, have proven to be a more suitable alternative material.

To mitigate the harmful effects of alkaline metals that can be introduced by using OSHL and RPG as raw materials, it is necessary to treat the recycled materials. Alkaline metals in the raw materials tend to volatilize under high temperatures, so heat treatment is employed to reduce their content. By adjusting the formulation of OSHL and RPG, the conditions that promote the thermal volatilization of alkaline metals can be identified. When the raw materials meet the standards for cement clinker production, the synthesized cement clinker and its hydration products are characterized using X-ray diffraction (XRD), thermogravimetric analysis (TGA), and scanning electron microscopy (SEM). This analysis aims to evaluate the feasibility of using waste glass and oyster shells as raw materials for cement production, from a microscopic perspective.

## 2. Materials and Methods

### 2.1. Selection and Treatment of Raw Materials

The primary raw materials used for the synthesis of cement clinker, namely, waste glass and oyster shells, were collected from Zhoushan, Zhejiang Province, China. The SiO_2_, Al_2_O_3_, and Fe_2_O_3_ were of analytical grade and were sourced from West Long Scientific. Prior to grinding, the waste glass and oyster shells were thoroughly washed using a water-soaked brush to remove any surface residues. The cleaned samples were then dried in an oven at 105 °C for 48 h. Subsequently, an impact crusher (China Haisheng Instrument Factory DE-4, Shaoxing, China) was employed to pulverize the shells. The resulting fine powders were sieved to a particle size of 100 μm to prepare recycled powdered glass (RPG) and oyster shell powder (OSHL). The chemical composition of OSHL was determined using energy-dispersive X-ray spectroscopy (EDX), X-ray diffraction (XRD), and thermogravimetric analysis (TGA). The main component of RPG is SiO_2_ (65%–75%), followed by NaO (12%–15%), CaO (6%–12%), and a small amount of Al_2_O_3_ (0.5%–5%) [9]. Excess alkaline oxides from the glass can cause flash setting in the produced cement; therefore, heat treatment is employed to reduce the alkaline oxides in the clinker.

#### 2.1.1. Quantification of OSHL

**Figure 1:**
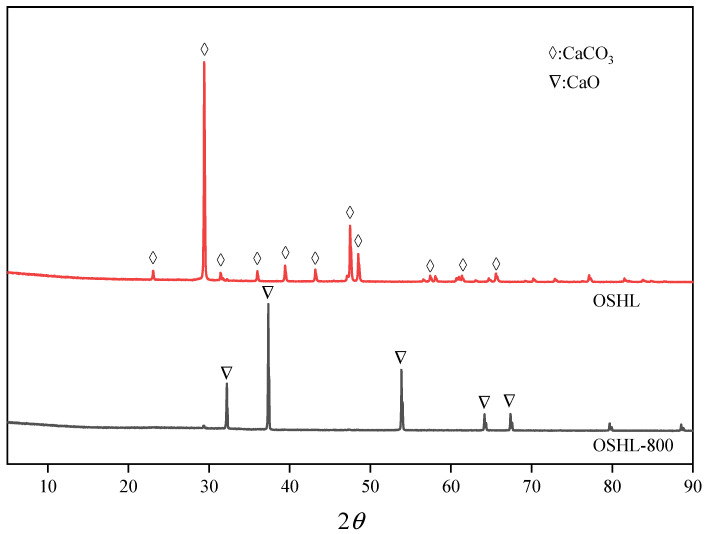
X-ray diffraction spectra of OSHL and OSHL-800.

Qualitative and quantitative experiments were conducted on OSHL. EDX (Oxford Instruments AZtec X-MaxN 80 energy-dispersive X-ray spectrometer, Oxfordshire, UK) and XRD analyses (Rigaku Ultima IV X-ray diffraction analysis, Tokyo, Japan) were performed on both OSHL and oyster shell powder treated at 800 °C (OSHL-800), as shown in Table 1 and Figure 1. The results indicate that the primary component of oyster shell powder is CaCO_3_. During the high-temperature calcination process, CaCO_3_ undergoes a decomposition reaction, resulting in the formation of CaO. This reaction can be represented as follows:

CaCO_3_→CaO + CO_2_ (800 °C high temperature)

This provides reliable evidence for the content of CaCO_3_ in OSHL, as determined by TGA.

**Figure 2:**
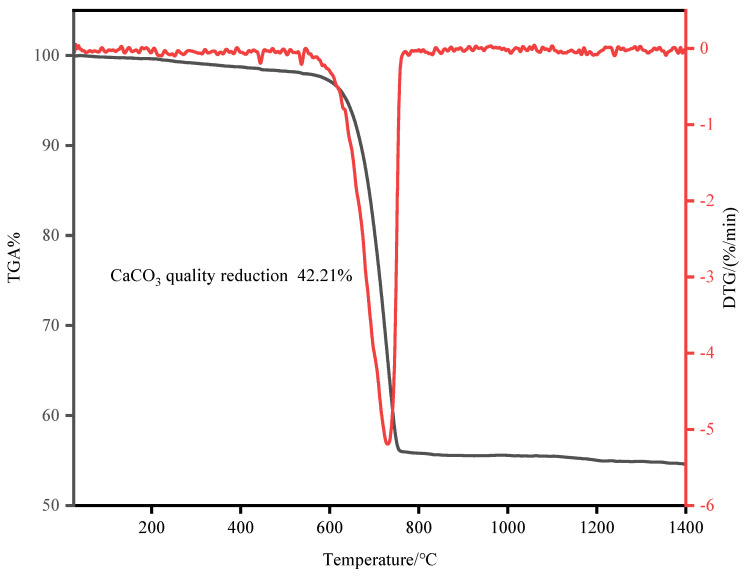
Thermogravimetric analysis of OSHL.

TGA analysis (NETZSCH STA449F3 simultaneous thermal analyzer, Selb, Germany) was conducted on OSHL, yielding the results presented in Figure 2. The analysis was performed over a temperature range from 25 °C to 1400 °C, with a heating rate of 10 °C per minute in a nitrogen atmosphere, at a gas flow rate of 200 mL per minute.

From the TGA curve, it is evident that significant mass loss occurs between 570 °C and 760 °C, reaching 42.21% [10]. This mass loss is primarily attributed to the decomposition of CaCO_3_ into CaO, releasing CO_2_.

Given the mass loss of 42.21%, the content of CaCO_3_ can be calculated using the following formula:

$${\mathrm{CaCO}}_{3}\;(\%)=\frac{\Delta m}{44}\times 100$$

$${\mathrm{CaCO}}_{3}\;(\%)=\frac{42.21}{44}\times 100=95.93\%$$

This result indicates that the calcium carbonate content in the OSHL is as high as 95.9%.

#### 2.1.2. Raw Material Thermal Treatment

The primary purpose of the raw material’s thermal treatment is to reduce the content of alkaline oxides in the raw materials. High-temperature treatment effectively decomposes and removes some undesirable components, particularly excessive alkali metal oxides, thereby minimizing their potential negative impact on the performance of the cement. This process not only enhances the stability of the raw materials but also provides a higher-quality raw material foundation for subsequent cement clinker synthesis. Specific cooperation is shown in Table 2.

**Figure 3:**
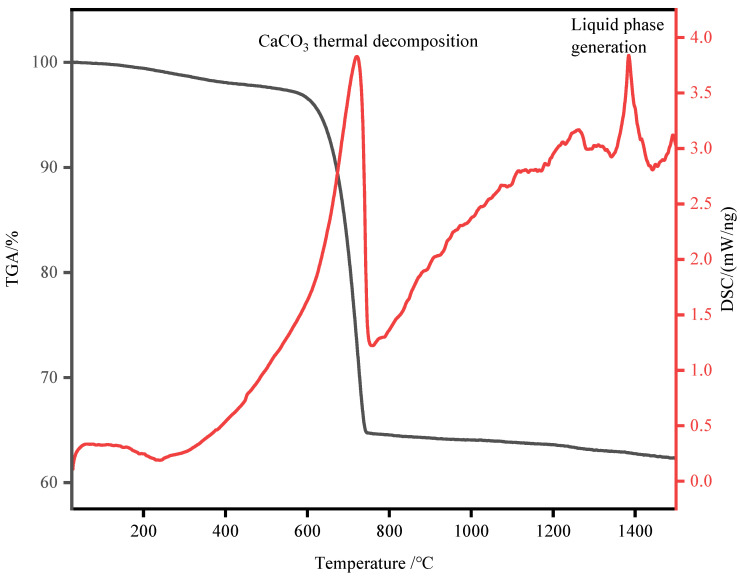
TGA of RPG and OSHL.

TGA analysis was conducted on a mixture sample of SHL and RPG in a 3.3:1 ratio, the results of which are presented in Figure 3. The analysis was performed over a temperature range from 25 °C to 1500 °C, with a heating rate of 10 °C per minute, in an air atmosphere, and with a gas flow rate of 200 mL per minute.

Through TGA, the main temperature points present during the calcination of recycled cementitious material raw materials were identified. Therefore, subsequent studies will focus on recycled cementitious materials calcined at temperatures of 800 °C, 1000 °C, 1200 °C, and 1400 °C, with a heating rate of 10 °C/min and a holding time of 30 min. The experimental combinations are outlined as follows.

Elemental analysis at different temperatures was conducted using EDX, yielding the results shown in Table 3. The sodium (Na) content significantly decreases with increasing temperature (H8 at 3.14% and H14 at 1.13%), indicating that Na volatilizes at high temperatures, although there is little variation seen between 1200 °C and 1400 °C. Research indicates that approximately 50% of alkali metals volatilize between 800 °C and 1000 °C during the calcination of cement kiln raw materials [11].

An analysis of samples H8, H10, and H12 revealed a direct linear relationship between the density of alkali-containing raw materials and the degree of volatilization [12]. However, within the high-temperature range of 1200 °C to 1400 °C, there is no significant difference in the volatilization rate of alkali metals, suggesting that the volatilization rate remains relatively stable in this temperature range, due to the formation of a liquid phase in the raw materials. Following treatment at 1200 °C, the Na content in the raw materials decreases by 70%. As Na is the primary alkali metal in the raw materials, this further indicates that the thermal treatment of the raw materials has achieved the desired effect.

By setting different mix ratios, the influencing factors of raw material thermal treatment were observed. The specific combinations are shown in Table 4.

Table 5 presents an elemental composition analysis of the samples under different thermal treatment conditions. A quantitative analysis of the elemental content of each sample was conducted using EDX at varying mix ratios. The studied samples, H12, H12-1, and H12-2, were obtained after calcination at 1200 °C.

The EDX analysis results indicate that alkali metals exhibit significant volatility under calcination conditions of 1200 °C for samples H12, H12-1, and H12-2. The OSHL contains trace amounts of Cl, which exhibits high volatility; its volatilization rate approaches 100% at temperatures above 800 °C, and Cl promotes the volatilization of alkali metals. In a comparison between H12 and H12-1, it is evident that the thermal volatilization rate of alkali metals is significantly increased in the presence of trace Cl [8].

Additionally, the introduction of chemically pure Al_2_O_3_ and Fe_2_O_3_ in samples H12 and H12-2 appears to suppress the volatilization of Na, likely due to the presence of aluminum and iron oxides, which may reduce Na volatility to some extent. However, the mechanisms and patterns of this phenomenon remain inadequately explored and require further experimental investigation.

Currently, it is widely accepted that the contents of alkali metals in cementitious materials should be controlled to remain below 1%. The alkali oxide content in sample H12 did not meet this requirement; therefore, by reducing the proportion of RPG in the raw materials, the alkali oxide content in sample H12-3, when tested after high-temperature calcination, meets the specifications for cementitious materials.

### 2.2. Methods

#### 2.2.1. Experimental Design

The RPG and OSHL were subjected to heat treatment at 1200 °C to ensure that their alkali metal content complies with cement raw material standards. Subsequently, based on the chemical composition of each raw material, the Bogue equation was used to calculate the mixing ratio of chemical pure ecological cement (CEC) and the RPG and OSHL ecological cement (ROEC) [13]. This empirical formula, being based on stoichiometric ratios, has been widely applied in the cement industry. By optimizing the mixing ratios, the mineral composition of the cement can be improved, thereby enhancing its performance [14]. The specific mixing ratios are shown in Table 6. CaSO₄•2H_2_O acts as a mineralizer in the cement’s raw materials, which can lower the synthesis temperature of CEC.

#### 2.2.2. Synthesis

The raw materials, RPG and OSHL, were placed in a muffle furnace for heat treatment at 1200 °C for 30 min at a 1:3.3 ratio. Subsequently, the mixture was placed in a platinum crucible according to the respective proportions and synthesized at 1400 °C for 30 min. C3S and C2S are highly sensitive to cooling temperature; if the cooling rate of the cement clinker is too slow, the crystal form of C2S can transform into γ-C2S during the hydration reaction, creating a non-reactive phase [15]. Therefore, after removing the clinker, which is at 1400 °C, from the furnace, it is essential to immediately cool it to room temperature (20 °C) using a portable fan while it is still in the platinum crucible.

#### 2.2.3. Milling

**Figure 4:**
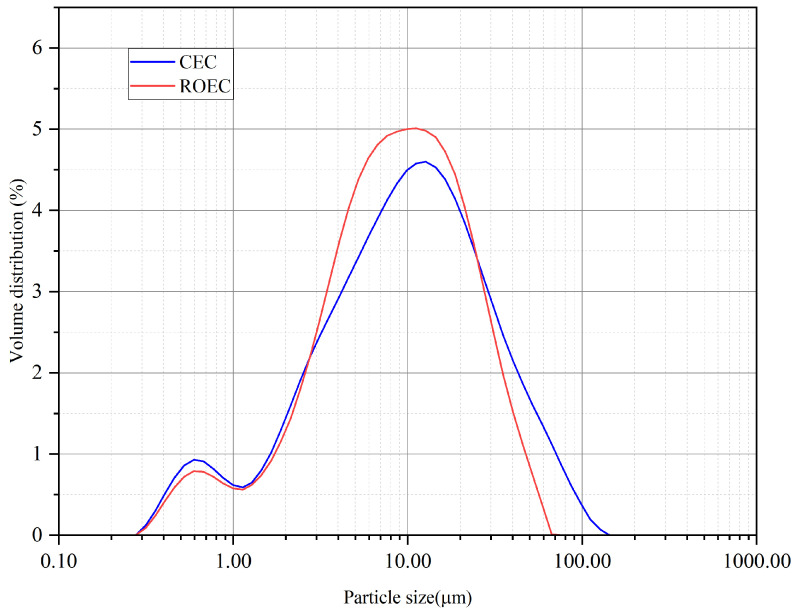
CEC and ROEC particle size distribution.

The workability and strength development of cement-based materials depend not only on the particle size of the cement but also significantly on the particle size distribution. The synthesized cement clinker was first crushed manually and then ground down using an impact grinder (Haisheng Instruments DF-4, Shaoxing, China) for 2 min. Gypsum (Xilong Technology, Shantou, China) (3 wt.%) was added prior to grinding. The material was sieved to a particle size of 100 μm (150 mesh) and then analyzed for particle size distribution, using a laser diffraction instrument (Malvern Mastersize 2000, Worcestershire, UK), as shown in Figure 4.

#### 2.2.4. Hydration

For sample preparation, the cement was mixed with distilled water at a water-to-cement mass ratio of 0.5:1, using manual stirring for 10 min. The mixture was then poured into molds measuring 5 × 5 × 5 mm^3^ and cured in a constant-temperature chamber (25 °C, relative humidity 95%). After 24 h, the specimens were de-molded and placed back into the constant-temperature chamber for further curing.

#### 2.2.5. Characterization Method

EDX: Elemental analysis was performed using an energy-dispersive X-ray spectrometer (Oxford AZtec X-MaxN 80, Oxfordshire, UK). A small amount of sample powder (approximately 3 mg) was placed on the stage of a high-vacuum ion sputter coater (Quorum, East Sussex, UK) and coated with a uniform layer of 5 nm-thick gold nanoparticles. The gold-coated sample powder was then evenly dispersed on a sample holder with conductive adhesive, and, after ensuring conductivity, testing commenced. The weight percentage (wt.%) values were analyzed from measurements taken at three different points.

XRD: X-ray diffraction analysis was conducted using an Ultima IV X-ray diffractometer (Rigaku Corporation, Tokyo, Japan) (Cu Kα1, wavelength 1.54184 Å, operating conditions 40 kV, and 30 mA). Data collection was performed in the 2θ range of 5° to 90°, with a step size of 0.02° and a time interval of 1.5 s.

SEM: Scanning electron microscopy was performed using a Tescan Mira 3 XH field emission scanning electron microscope (Quorum, East Sussex, UK). The SEM system was started and an appropriate accelerating voltage (10–20 kV) was selected to ensure that the system reached the required vacuum level. The gold-coated sample was fixed on the sample stage and positioned. The focus and image parameters were adjusted to ensure clarity, and the required images and data were saved. Finally, the electron beam was turned off, the sample was removed, and the equipment was shut down.

## 3. Results and Discussion

### 3.1. Characterization of Synthesized Anhydrous Clinker

The particle size distribution of recycled clinker significantly influences the reactivity and mechanical properties of cementitious materials. Therefore, laser particle size analysis was performed on the ground-down and screened samples of the recycled clinker. Figure 4 shows the particle size distribution of CEC and ROEC.

The particle size distribution of the ROEC samples closely resembles that of the CEC, with a peak near 80 μm. Although RPG was introduced into the ROEC samples, the particle size distribution remained similar to that of the CEC samples. This indicates that the introduction of RPG did not significantly alter the particle size distribution characteristics of the materials, demonstrating consistency during the production and grinding processes.

**Figure 5:**
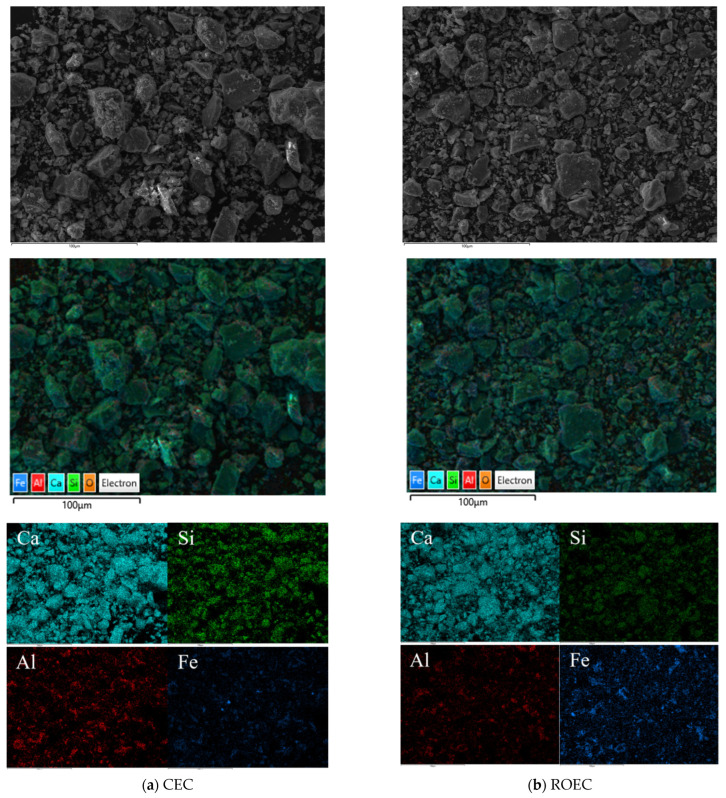
Distribution of the CEC and ROEC elements.

In actual production, the manufacturing of commercial cementitious materials occurs under dynamic conditions using a rotary kiln, while the regenerated cementitious materials synthesized at the laboratory scale are produced under static conditions in a muffle furnace. To understand the morphological differences between dynamic and static synthesis conditions, the microstructure of the clinker was examined, as shown in Figure 5. According to EDX elemental mapping, aluminum and iron ions are typically distributed between C3S crystals in the form of C3A or C4AF. Thus, the morphology and size of the anhydrous crystalline phase are influenced by manufacturing process conditions (dynamic or static). Therefore, the characteristics of synthesized regenerated cementitious materials may differ from those of commercial cementitious materials [16].

In this experiment, CEC serves as the control group, while ROEC is the experimental group, in which RPG and OSHL are introduced to replace a portion of the traditional calcium-based raw materials. The elemental contents of CEC and ROEC are shown in Table 7. By comparing the elemental contents of the two groups of materials, the success of the experimental group of regenerated cementitious materials can be evaluated, confirming that the contents of alkaline metal oxides have been reduced to a reasonable level.

**Figure 6:**
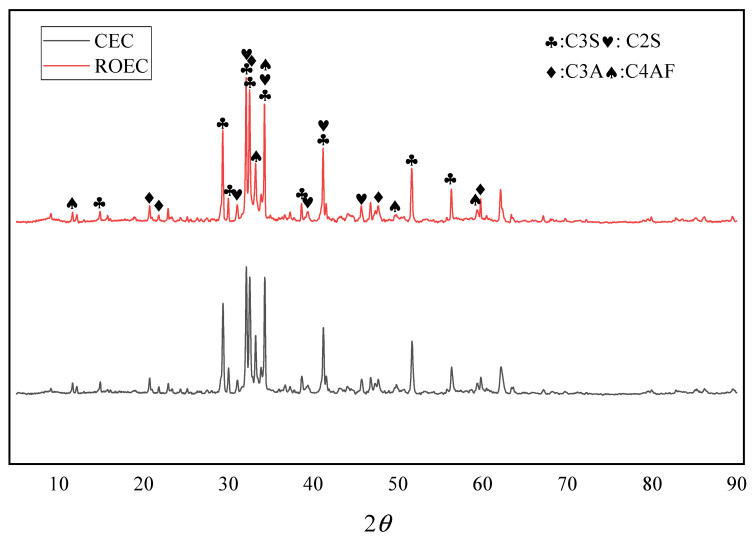
XRD patterns of CEC and ROEC.

The XRD spectra analysis of CEC and ROEC is shown in Figure 6. The diffraction peaks of CEC and ROEC are nearly identical, both displaying typical mineral phases such as C3S, C2S, C3A, and C4AF. C2S can exist in various forms, including α-C2S, β-C2S, and γ-C2S, at different temperatures [17]. During the production process of commercial cementitious materials, rapid cooling is typically employed in the β phase to prevent the transformation of α-C2S and β-C2S into the γ-C2S phase [18]. The reflection peaks observed at 2θ values of 29° and 48° correspond to the trigonal crystalline CaCO_3_ present in the raw materials, which disappear significantly after calcination at temperatures above 800 °C. Additionally, the clear X-ray reflections associated with anhydrous cement phases (such as C3S, C2S, C3A, and C4AF) are observed in the range of 2θ from 28.5° to 35°. These results indicate that all CaCO_3_ in OSHL has decomposed and completely reacted with the decomposition products of RPG, resulting in the formation of four primary cement phases, which is similar to the reaction process of limestone. According to the XRD results, the sintering process at 1400 °C facilitates the reaction between residual lime and C2S to synthesize C3S.

### 3.2. Hydration Microstructural Characterization

The hydration process significantly affects the performance of cement-based materials, making it crucial to characterize the microstructure of hydration products. In this experiment, SEM, EDX, and XRD were used to study the micro-morphology and elemental composition of the hydration products.

Table 8 presents the elemental composition of cement hydration products after curing for 7 and 60 days. Due to differences in the raw materials, the ROEC contained Na and Mg elements after hydration. The hydration products of both ROEC and CEC were examined after 7 and 60 days of curing, and the curing time for the C element increased; the reasons for this phenomenon will be discussed in subsequent analyses.

**Figure 7:**
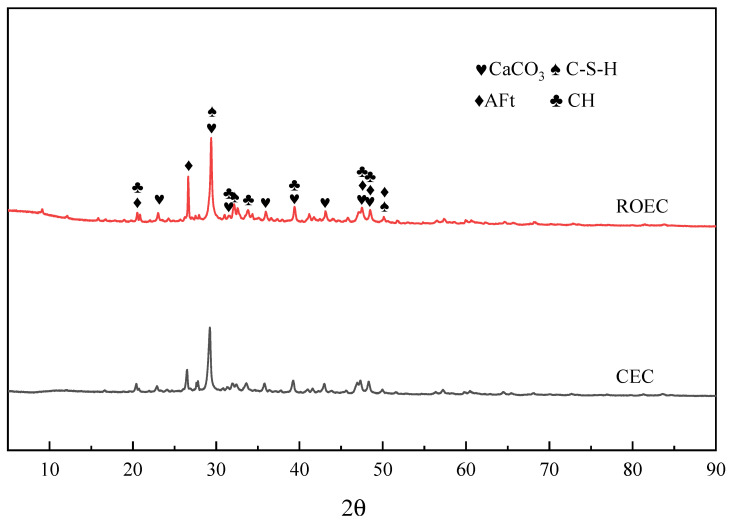
XRD patterns of CEC and ROEC hydration products after 7 days of curing.

Figure 7 shows the XRD patterns of the cement hydration products after 7 days of curing. At this stage, the hydration products of ROEC and CEC were similar; however, the peak intensity of the AFt phase in ROEC was significantly higher than that in CEC. The accelerating effect of alkaline ions (such as Na⁺ and K⁺) in the cement hydration reaction operates through several mechanisms: first, the dissolution of alkaline ions increases the pH of the cement paste. In a high-pH environment, the dissolution rates of C_3_S and C_2_S are enhanced, which accelerates the formation of C-S-H and calcium hydroxide (CH, Ca(OH)_2_), thereby significantly improving the early strength of the cement. Additionally, the highly alkaline environment promotes the formation of AFt [16]. Under these alkaline conditions, calcium aluminate compounds (such as C_3_A) react with sulfate ions to form AFt, particularly during the early hydration stage. This increase in pH accelerates the dissolution of the aluminum phase, allowing aluminum ions to more rapidly combine with sulfate and calcium ions to generate AFt [19].

**Figure 8:**
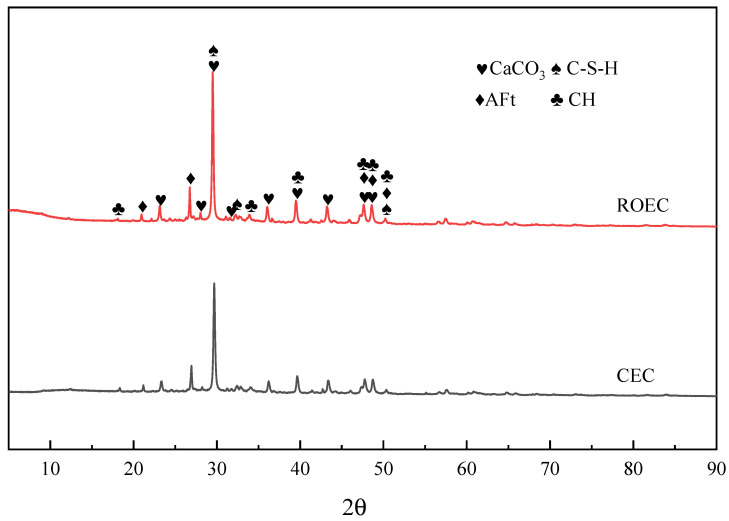
XRD patterns of CEC and ROEC hydration products after 60 days of curing.

Figure 8 shows the XRD patterns of the cement hydration products after 60 days of curing. At this stage, the hydration products of ROEC and CEC are largely similar, with calcium carbonate (CaCO_3_) being the main hydration product. The presence of calcium carbonate is attributed to the atmospheric carbonation of the sample surface. When C_3_S and β-C_2_S undergo carbonation, calcite is the predominant crystal form of CaCO_3_, with crystal sizes of less than 1 μm [10]. Additionally, when the γ-C_2_S carbonates, the main crystal forms of the resulting calcium carbonate are calcite and vaterite [20].

**Figure 9:**
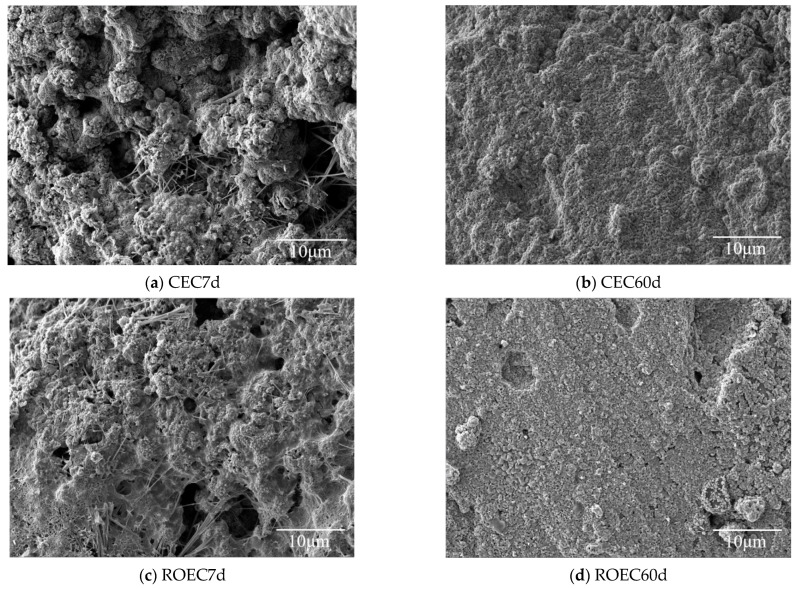
Electron microscopy images of CEC and ROEC hydration products cured for 7 and 60 days.

Figure 9 shows the SEM images of the hydration products of CEC and ROEC after 7 and 60 days of curing, allowing for an analysis of the evolution of microstructure and the formation of hydration products [21]. At day 7 of the hydration reaction, both CEC and ROEC exhibit sufficient hydration, with similar C-S-H morphologies. The C-S-H gel is the main hydration product and plays a crucial role in enhancing the mechanical properties of the cementitious materials [22]. Its nucleation and growth can be facilitated by calcium ions and silicate ions that are concentrated in the cement paste. CH, CaCO_3_, and AFt are present in both hydration products, with ROEC showing more complete hydration, as alkaline ions (such as Na⁺ and K⁺) accelerate the cement hydration reaction. By day 60, the surfaces of the CEC and ROEC hydration specimens appear more uniform, with a significant increase in the number of granular and needle-like hydration products, which are evenly distributed. Surface XRD analysis indicates that the predominant phase on the surface is calcite, and the morphologies of hydration products for CEC and ROEC exhibit similarities over the long term.

## 4. Conclusions

This study synthesized cement clinker using RPG and OSHL as primary raw materials, validating the hypothesis that the high alkaline metal oxide content in RPG and the limestone-like chemical composition of OSHL can jointly produce clinker that meets modern cement production standards while reducing alkaline metal oxide levels. The results demonstrate that this synthesis pathway not only enhances the physicochemical properties of clinker but also provides a new solution for resource utilization and sustainable development in the cement industry. The main findings and extended analysis are as follows:

(1) Key Findings Validating the Hypothesis

The study shows that adjusting the heat treatment conditions (e.g., 1200 °C) and introducing trace amounts of chlorine significantly improve the volatilization efficiency of alkaline metals, resulting in the clinker meeting the required standards for alkaline metal oxide content. The mineral phases of clinker synthesized from RPG and OSHL (C3S, C2S, C3A, and C4AF) are consistent with those of traditional clinker, confirming the feasibility and stability of these new raw materials.

(2) Performance Enhancement and Engineering Potential

During hydration, ROEC exhibits higher reactivity, significantly improving its early strength. In particular, the presence of alkaline metal ions further accelerates the hydration reaction, making ROEC suitable for those applications requiring rapid strength development, such as high-strength concrete and quick repair materials.

(3) Resource Utilization and Sustainability

The proposed process provides a practical solution for the high-value utilization of industrial waste, such as RPG, while reducing its dependence on natural limestone resources. This contributes both theoretical and practical support for the green transformation of the cement industry.

However, this study has limitations: it is limited to laboratory-scale experiments and lacks validation under industrial production conditions; long-term performance aspects, such as durability and volume stability under extended curing conditions, remain insufficiently studied; and a systematic evaluation of carbon emissions and environmental impacts during production is absent.

Future research should focus on large-scale industrial trials to verify the raw material’s adaptability and process feasibility, further explore the performance evolution of clinker under long-term curing, and conduct full lifecycle assessments to clarify energy consumption and carbon emissions. These efforts will provide a solid foundation for green certification and sustainable development. This study offers theoretical insights and practical references for utilizing waste materials and advancing the green transformation of the cement industry.

## Figures and Tables

**Table 1:** Elemental composition of OSHL and OSHL-800, as measured by EDX.

Element (wt.%)	O	C	Na	Mg	Al	Si	S	Cl	Ca	Fe
OSHL	33.87	8.59	0.55	0.1	0.06	0.06	0.14	0.36	55.88	0.39
OSHL-800	24.6	2.28	0.5	0.22	0.06	0.08	0.17	0.29	71.52	0.28

**Table 2:** Calcination temperature for RPG and OSHL.

No.	Mix Ratio (Mass Ratio)	Calcination Temperature (°C)
H8	RPG:OSHL = 1:3.3	800
H10	RPG:OSHL = 1:3.3	1000
H12	RPG:OSHL = 1:3.3	1200
H14	RPG:OSHL = 1:3.3	1400

**Table 3:** Element contents of RPG and OSHL after heat treatment.

Element (wt.%)	C	O	Na	Mg	Al	Si	S	Cl	Ca	Fe
H8	2.51	35.18	3.14	0.57	0.48	9.58	0.49	0.09	47.13	0.37
H10	2.47	34.76	2.31	0.47	0.52	9.67	0.21	0	47.91	0.37
H12	2.24	34.38	1.03	0.41	0.38	9.87	0.14	0	48.65	0.41
H14	2.14	33.81	1.13	0.33	0.47	10.14	0.11	0	48.85	0.44

**Table 4:** Heat treatment mix ratio for RPG and OSHL.

No.	Mix Ratio (Mass Ratio)	Calcination Temperature (°C)
H12	RPG:SHL = 1:3.3	1200
H12-1	RPG:CaCO_3_ = 1:3.3	1200
H12-2	RPG:SHL:Al_2_O_3_:Fe_2_O_3_ = 1:3.3:0.14:0.06	1200

**Table 5:** Element contents of RPG and OSHL after heat treatment.

Element (wt.%)	C	O	Na	Mg	Al	Si	S	Cl	Ca	Fe
H12	2.24	34.38	1.03	0.41	0.38	9.87	0.14	0	48.65	0.41
H12-1	2.37	34.99	1.65	0.48	0.35	10.06	0.13	0	48.89	0.49
H12-2	2.04	33.39	2.12	0.45	3.62	9.61	0.13	0	46.94	1.63

**Table 6:** Mixing ratio of CEC and ROEC raw materials.

Code	Mixing Ratio (Weight Ratio)
CEC	SiO_2_:CaCO_3_:Al_2_O_3_:Fe_2_O_3_:CaSO_4_•2H_2_O = 1:5.23:0.246:0.154:0.01
ROEC	RPG:OSHL:Al_2_O_3_:Fe_2_O_3_ = 1:3.3:0.14:0.06

**Table 7:** Contents of CEC and ROEC elements.

Element (wt.%)	C	O	Na	Mg	Al	Si	S	Ca	Fe
CEC	3.92	34.06	0	0	1.45	8.48	1.12	45.13	5.72
ROEC	2.39	34.33	0.35	0.15	1.96	8.08	1.39	45.25	5.9

**Table 8:** CEC and ROEC hydration products: maintenance of element content for 7 and 60 days.

Element (wt.%)	C	O	Cl	Na	Mg	Al	Si	S	Ca	Fe
CEC7d	6.69	40.34	0	0	0	2.99	3.65	1.65	39.8	4.88
ROEC7d	7.85	40.5	0	0.45	0.15	3.12	3.35	1.73	38.9	3.65
CEC60d	7.21	39.13	0	0	0	2.85	3.87	1.54	40.83	4.57
ROEC60d	8.39	41.12	0	0.33	0.12	3.12	3.29	0.74	39.05	3.54

## Data Availability

The original contributions presented in the study are included in the article, further inquiries can be directed to the corresponding author/s.
